# Risk factors for the development of esophageal candidiasis among patients in community hospital

**DOI:** 10.1038/s41598-021-00132-w

**Published:** 2021-10-19

**Authors:** Hideyuki Ogiso, Seiji Adachi, Masatoshi Mabuchi, Yohei Horibe, Tomohiko Ohno, Yusuke Suzuki, Osamu Yamauchi, Takao Kojima, Eri Takada, Midori Iwama, Koshiro Saito, Takuji Iwashita, Takashi Ibuka, Ichiro Yasuda, Masahito Shimizu

**Affiliations:** 1 Department of Gastroenterology/Internal Medicine, Gihoku Kosei Hospital, 1187-3 Takatomi, Yamagata, 501-2105 Japan; 2 Department of Gastroenterology, Gifu University Graduate School of Medicine, Gifu, Japan; 3 Third Department of Internal Medicine, University of Toyama, Toyama, Japan

**Keywords:** Gastroenterology, Risk factors

## Abstract

The aim of this study was to clarify risk factors for esophageal candidiasis (EC) in immunocompetent patients in a community hospital. 7736 patients who underwent esophagogastroduodenoscopy at our hospital from April 2012 to July 2018 were enrolled. The relationships between EC and the following factors: age, gender, body mass index, lifestyle, lifestyle-related diseases, medication, and endoscopic findings were analyzed. EC was observed in 184 of 7736 cases (2.4% morbidity rate). Multivariate analysis revealed that significant risk factors for the development of EC were: diabetes mellitus {odds ratio (OR): 1.52}, proton pump inhibitor (PPI) use (OR: 1.69), atrophic gastritis (AG) (OR: 1.60), advanced gastric cancer (OR: 4.66), and gastrectomy (OR: 2.32). When severe EC (Kodsi grade ≥ II) was compared to mild EC (grade I), the most significant risk factors were advanced gastric cancer (OR: 17.6) and gastrectomy (OR: 23.4). When considering the risk of AG and PPI use with EC development, the risk increased as follows: AG (OR: 1.59), PPI use (OR: 2.25), and both (OR: 3.13). PPI use, AG, advanced gastric cancer and post-gastrectomy are critical risk factors for the development of EC. We suggest close monitoring for EC development when PPIs are administered to patients with these factors.

## Introduction

Esophageal candidiasis (EC) is the most common esophageal infection. It is caused by candida fungi that are normally present in the oral cavity and digestive tract^1^. *Candida albicans* (*C. albicans*), the most important fungal pathogen in humans, is a gastrointestinal commensal that forms colonies on the esophagus in up to 20% of people^2^. The incidence of EC is 0.3–5.2% in the general population^3^, but is much higher in patients with severe immunodeficiency, such as human immunodeficiency virus (HIV)-infected patients^4^. A study by Takahashi et al.^5^ found that the prevalence of EC among patients who underwent esophagogastroduodenoscopy in their cohort was 1.7% overall, but was 9.8% in patients with HIV infection. Although most EC cases are asymptomatic and discovered by chance during endoscopic examinations, some cases can cause symptoms such as painful swallowing, chest discomfort, bleeding ulcers, and stenosis with passage disorders.

Various co-morbidities are associated with EC including: diabetes mellitus, steroid or immunosuppressant use, malignant tumors, and acquired immune deficiency syndrome (AIDS) caused by HIV infection^6^. Proton pump inhibitors (PPIs), which are widely used for peptic ulcers, gastroesophageal reflux disease (GERD), and eradication therapy against *Helicobacter pylori* (*H. pylori*), have been reported to increase the risk of developing EC^7–9^. Importantly, EC is observed after PPI administration even in patients without underlying diseases that cause immunosuppression^10^. PPI can reduce gastric acid secretion, resulting in a state of achlorhydria in the stomach^11^ and the low-acid environment created by PPIs may affect the body’s antifungal activity against Candida.

Gastric and esophageal pathologies are often associated with the secretion dynamics of gastric acid. Esophageal hiatal hernia and GERD cause the backflow of gastric acid into the esophagus, increasing the acidity. Conversely, gastrectomy and atrophic gastritis caused by *H. pylori* infection reduces gastric acid secretion decreasing the acidity in the esophagus. Therefore, any pathology that affects the acid level in the esophagus may greatly influence the development of EC. However, the correlations between endoscopic findings regarding gastric and esophageal pathologies and EC have not been fully characterized.

The purpose of this study was to examine risk factors for the development of EC among immunocompetent patients in a community hospital, focusing on associations with gastric and esophageal pathologies. In addition, predictors for the development and severity of EC in these patients were also evaluated using medical records.

## Methods

### Study design

Patients who underwent esophagogastroduodenoscopy at Gihoku Kosei Hospital between April 2012 and July 2018 were retrospectively analyzed. They included both of inpatients and outpatients. Among the 18,153 procedures, 7736 patients were retained, after excluding duplications, and were included in this study. In the 7736 patients, 4271 had abdominal symptoms such as upper abdominal pain and 3465 were screened for gastric cancer. Patient histories (age, gender, lifestyles, comorbidities, and medication) prior to examination were recorded. This study protocol was approved by the Institutional Review Board of the Gihoku Kosei Hospital, Gifu, Japan (Reference No. 20200801). Informed consent was obtained from all patients before total colonoscopy examination for clinical research. The study was carried out in accordance with the Declaration of Helsinki.

EC was diagnosed based on typical endoscopic findings or pathological findings. Biopsy was conducted to distinguish EC from other diseases, such as cytomegalovirus infection, herpes simplex virus infection, and eosinophilic or inflammatory lesions. The Kodsi classification was used to categorize EC severity^12^. To assess gastric atrophy, the Kimura-Takemoto classification was applied^13^.

### Statistical methods

Statistical analysis was performed using EZR^14^. The Pearson χ^2^ test was used for categorical data to compare the proportions of each candidate risk factor. Fisher’s exact test was applied to the categorical data when one of the categorical numbers was less than 10. Multivariate logistic regression analysis was used to analyze the independent risk factors for the development and severity of EC. Values of *P* < 0.05 were considered statistically significant.

### Ethics

All subjects provided written informed consent before enrollment. The study protocol was approved by the Ethics Committee of Gihoku Kosei Hospital (Reference No. 20200801) and adhered to the tenets of the Declaration of Helsinki.

## Results

### Characteristics of patients

The characteristics of the 7736 patients were as follows: median age: 61.3 years (range, 15–105 years), gender: 3929 men (50.8%), and median body mass index (BMI): 23.0 kg/m^2^ (range, 10.7–37.3). Diabetes mellitus was found in 860 patients (11.1%). PPIs were taken by 1253 patients (16.2%). EC was observed in 184 of 7736 patients (2.4% incidence); this rate was similar to a previous report of regional general hospitals^10^. Moreover, 3161 patients had atrophic gastritis (40.9%) (Table 1).

**Table 1:** Clinical characteristics of study patients (n = 7736).

Factor	Number (n) or mean^a^	Rate (%) or range^b^
Age (years)	61.3^a^	15–105^b^
Sex (M/F)	3929/3807	50.8/49.2
Body mass index	23^a^	10.7–37.3^b^
**Preference history**		
Alcohol consumption	3188	41.2
Smoking	1360	17.6
**Life-style diseases**		
Diabetes mellitus	860	11.1
Hypertension	2378	30.7
Dyslipidemia	1195	15.4
**Current medication**		
PPI	1253	16.2
H_2_-blocker	399	5.2
NSAIDs	884	11.4
Oral steroid use	91	1.2
Inhaled sterid use	50	0.6
**Endoscopic findings**		
Esophageal candidiasis	184	2.4
GERD	399	5.2
Esophageal hiatal hernia	658	8.5
Atrophic gastritis	3161	40.9
Advanced gastric cancer	115	1.5
Postgastrectomy	181	2.3

*PPI* proton pump inhibitor, *NSAID* non-steroidal anti-inflammatory drug, *GERD* gastroesophageal reflux disease. ^a^median, ^b^range.

### Predictors for the development of EC

Univariate analysis showed that age over 65 years {odds ratio (OR): 1.95; *P* < 0.001), diabetes mellitus (OR: 1.85; *P* = 0.003), PPI use (OR: 2.2; *P* < 0.001), oral steroid use (OR: 2.96; *P* = 0.021), inhaled steroid use (OR: 3.62; *P* = 0.031), atrophic gastritis (OR: 1.63; *P* = 0.001), advanced gastric cancer (OR: 5.04; *P* < 0.001), and gastrectomy (OR: 2.48; *P* = 0.010) were significant risk factors for EC. BMI over 25 kg/m^2^ (OR: 0.51; *P* = 0.002) was independently associated with a decreased risk of EC. In contrast, GERD (OR: 0.83; *P* = 0.737) and esophageal hiatal hernia (OR: 0.55; *P* = 0.081) were not significant risk factors in this study (Table 2).

**Table 2:** Univariate analysis of development of esophageal candidiasis.

Factor	Odds ratio	95% CI	*P* value
Age (> 65)	1.95	1.41–2.71	< 0.001**
Sex (M)	1.31	0.96–1.79	0.085
Body mass index (≥ 25)	0.51	0.31–0.80	0.002**
**Preference history**			
Alcohol consumption	0.98	0.71–1.33	0.94
Smoking	0.69	0.43–1.08	0.116
**Life-style diseases**			
Diabetes mellitus	1.85	1.22–2.71	0.003**
Hypertension	1.15	0.83–1.58	1.149
Dyslipidemia	0.89	0.57–1.37	0.68
**Current medication**			
PPI	2.2	1.56–3.06	< 0.001**
H_2_-blocker	1.53	0.81–2.68	0.128
NSAIDs	0.36	0.66–2.52	0.364
Oral steroid use	2.96	1.04–6.84	0.021*
Inhaled sterid use	3.62	0.94–10.1	0.031*
**Endoscopic findings**			
GERD	0.83	0.35–1.69	0.737
Esophageal hiatal hernia	0.55	0.24–1.07	0.081
Atrophic gastritis	1.63	1.20–2.22	0.001**
Advanced gastric cancer	5.04	2.48–9.42	< 0.001**
Gastrectomy	2.48	1.15–4.78	0.01*

*PPI* proton pump inhibitor, *NSAID* non-steroidal anti-inflammatory drug, *GERD* gastroesophageal reflux disease.

*P < 0.05; **P < 0.01.

**Figure 1:**
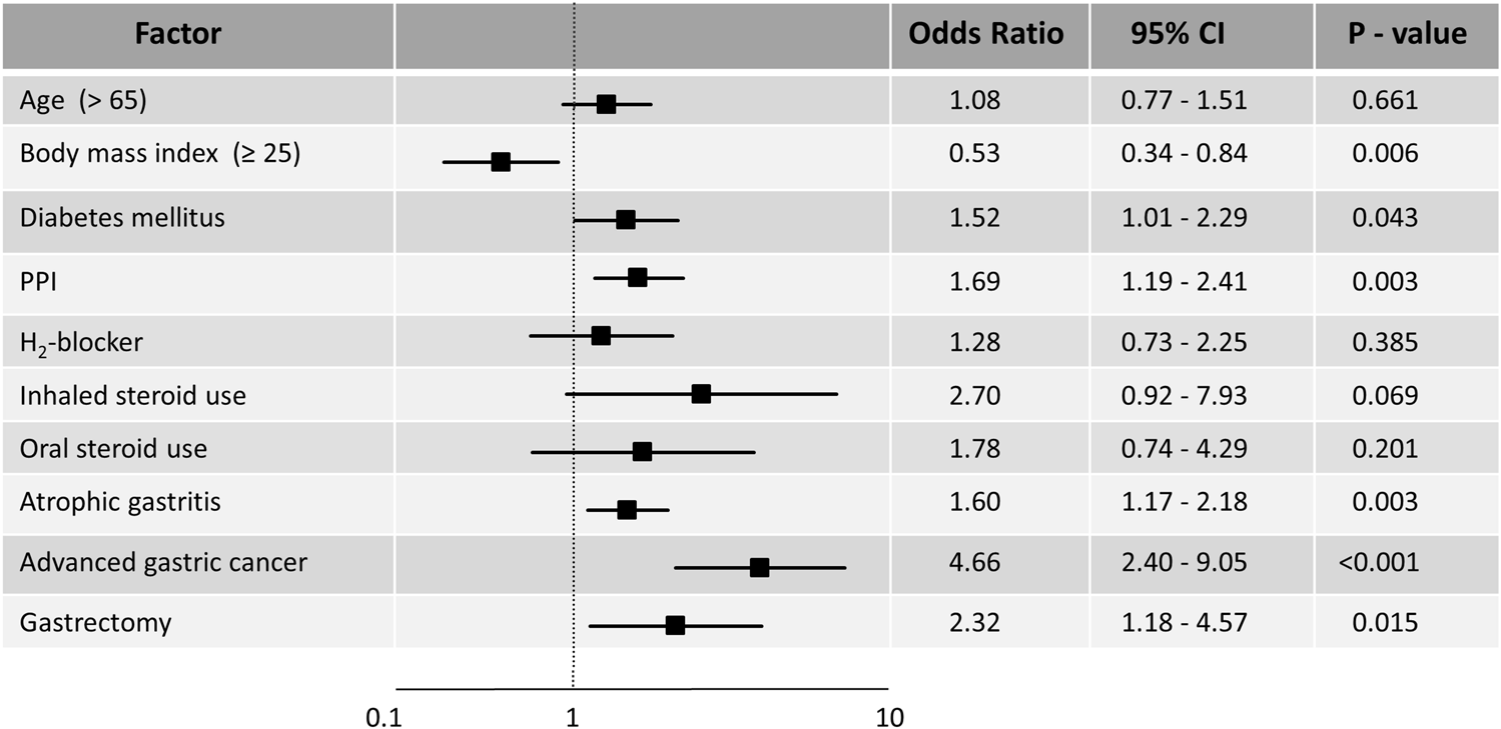
Predictors of esophageal candidiasis by a multiple logistic regression analysis. The plot shows the odds ratios (black squares) and 95% confidence intervals (CIs). *PPI* proton pump inhibitor.

Multivariate analysis also revealed that diabetes mellitus {OR: 1.52; 95% confidence interval (CI), 1.01–2.29, *P* = 0.043}, PPI use (OR: 1.69; 95% CI, 1.19–2.41, *P* = 0.003), atrophic gastritis (OR: 1.60; 95% CI, 1.17–2.18, *P* = 0.003), advanced gastric cancer (OR: 4.66; 95% CI, 2.40–9.05, *P* < 0.001), and gastrectomy (OR: 2.32; 95% CI, 1.18–4.57, *P* = 0.015) were independent risk factors for developing EC. In contrast, obesity (BMI ≥ 25) reduced EC risk (OR: 0.53; 95% CI, 0.34–0.84, *P* = 0.006). There were no significant differences between the two groups with regard to steroid use (inhaled: *P* = 0.069, oral: *P* = 0.201, respectively) (Fig. 1).

### Predictors of EC severity

**Figure 2:**
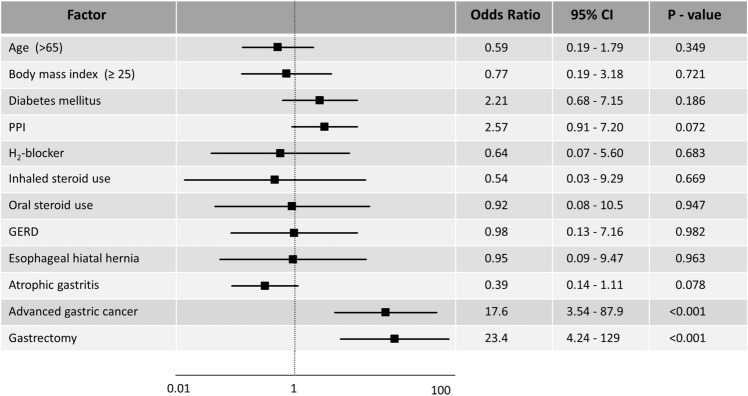
Predictors of disease severity of esophageal candidiasis by a multiple logistic regression analysis. The plot shows the odds ratios (black squares) and 95% confidence intervals (CIs) (horizontal lines). *PPI* proton pump inhibitor, *GERD* gastroesophageal reflux disease.

EC severity was evaluated using the Kodsi classification^12^. The severity of EC in the 184 patients examined in our cohort were: 146 patients with Kodsi grade I (79.3%), 20 with grade II (10.9%), 16 with grade III (8.7%), and 2 with grade IV (1.1%). EC patients were stratified as mild (grade I) or moderate/severe (grade II and higher). Patients with moderate/severe EC were more likely to have advanced gastric cancer (*P* = 0.003) and gastrectomy (*P* = 0.006) than patients with mild EC (Table 3). In multivariate analysis, advanced gastric cancer (OR: 17.6; 95% CI, 3.54–87.9, *P* < 0.001) and gastrectomy (OR: 23.4; 95% CI, 4.24–129, *P* < 0.001) were also identified as significant risk factors for developing sever EC. Atrophic gastritis (*P* = 0.078) and PPI use (*P* = 0.072) were not associated with EC severity in the present study (Fig. 2).

**Table 3:** Comparison of clinical characteristics based on severity of esophageal candidiasis.

Factor	Group I (n = 146)	Groups II, III, and IV (n = 38)	*P* value
Age (years)	66.7 ± 13.2	71.4 ± 15.8	0.066
Sex (M/F)	81/63	24/14	0.467
Body mass index (≥ 25)	18 (12.5%)	3 (7.9%)	0.769
**Preference history**			
Alcohol consumption	62 (43.1%)	12 (31.6%)	0.265
Smoking	20 (13.9%)	4 (10.5%)	0.789
**Life-style diseases**			
Diabetes mellitus	25 (17.4%)	9 (23.7%)	0.36
Hypertension	51 (35.4%)	10 (26.3%)	0.338
Dyslipidemia	21 (14.6%)	5 (13.2%)	1
**Current medication**			
PPI	37 (25.7%)	15 (39.5%)	0.108
H_2_-blocker	13 (9.0%)	1 (2.6%)	0.307
NSAIDs	9 (6.3%)	2 (5.3%)	1
Inhaled sterid use	3 (2.1%)	1 (2.6%)	1
Oral sterid use	4 (2.8%)	1 (2.6%)	1
**Endoscopic findings**			
GERD	6 (4.2%)	2 (5.3%)	0.673
Esophageal hiatal hernia	7 (4.9%)	1 (2.6%)	1
Atrophic gastritis	87 (60.4%)	23 (60.5%)	1
Advanced gastric cancer	5 (3.5%)	7 (18.4%)	0.003**
Gastrectomy	4 (2.8%)	6 (15.8%)	0.006**

*PPI* proton pump inhibitor, *NSAID* non-steroidal anti-inflammatory drug, *GERD* gastroesophageal reflux disease.

**P < 0.01.

### Impact of PPI use on the risk of EC in patients with atrophic gastritis

As both PPIs and atrophic gastritis cause reduced acidity, their combination may have a much greater impact on EC development than either alone. Therefore, we next examined whether PPI use may increase the risk for developing EC in patients with atrophic gastritis. The enrolled patients were stratified into the following 4 groups: patients without atrophic gastritis who did not receive PPI (control group, n = 3967), patients with atrophic gastritis who did not receive PPI (gastritis group, n = 2516), patients without atrophic gastritis who received PPI (PPI group, n = 608), and patients with atrophic gastritis who did receive PPI (gastritis plus PPI group, n = 645). The incidence of EC in each group was 1.6% (control group), 2.6% (gastritis group), 3.6% (PPI group), and 5.0% (gastritis plus PPI group). The OR with respect to the control was 1.59 (95% CI, 1.12–2.25), 2.25 (95% CI, 1.38–2.25), and 3.13 (95% CI, 2.03–4.83) for the gastritis group, PPI group, and gastritis plus PPI group, respectively (Table 4).

**Table 4:** Impact of atrophic gastritis and PPI use on the development of esophageal candidiasis.

	EC/total (n)^a^	EC (%)^b^	Odds ratio	95% CI
Control group	65/3967	1.6	Reference	–
Gastritis group	65/2516	2.6	1.59	1.12–2.25
PPI group	22/608	3.6	2.25	1.38–2.25
Gastritis plus PPI group	32/645	5	3.13	2.03–4.83

*EC* esophageal candidiasis, *PPI* proton pump inhibitor.

^a^The EC shows the number of cases of esophageal candidiasis in each group, and the total number of cases in each group.

^b^The EC (%) shows the incidence in each group.

## Discussion

The results of the present study clearly showed that diabetes mellitus, advanced gastric cancer, and PPI use are risk factors for developing EC among immunocompetent patients in our community hospital. This was consistent with results of previous studies^6–10^. In addition, we have demonstrated the first evidence that atrophic gastritis and gastrectomy are significant risk factors for the development of EC in immunocompetent patients. Importantly, the risk of developing EC increased when patients with atrophic gastritis were treated with PPI.

PPI use, atrophic gastritis, and gastrectomy are strongly associated with the creation of low-acid environments in the stomach (or remaining stomach in the case of gastrectomy). Atrophic mucosa does not produce normal amounts of acid. Stomach acidity is neutralized to a pH level of approximately 7.0 with increasing degree of mucosal atrophy^15^. Gastric pH is also reported to increase to a pH of 4.0–6.0 after PPI administration^16^. Therefore, PPI use and atrophic gastritis both contribute to a low-acid environment in the stomach and esophagus, which may increase the pathogenicity of Candida fungi. *C. albicans* can switch to its infectious pathogenic hyphal form in low-acid environments^17^. Elevation of pH by vagotomy is also associated with overgrowth of *C. albicans* in the gastric lumen^18^.

In addition to suppressing the secretion of gastric acid, PPI administration may also reduce host antifungal immunological responses. Natural killer cell activity against *C. albicans* in the spleen was shown to be inhibited by the PPI omeprazole^19^. Macrophages use intracellular proton pumps to maintain the acidic environments of endosomes and lysosomes, essential for fungal killing^20,21^. These results suggest that PPI administration could directly decrease the ability of the immune system to protect against Candida infection.

In the present study, EC patients were less likely to have a high BMI (≥ 25); however, the precise mechanism for this finding is unclear. In other diseases, obesity is a risk factor for infection^22^. Certainly, obesity is associated with diabetes mellitus, which we have shown to be an independent risk factor for EC^6^. However, lower BMI is reported to be an independent risk factor of Candida colonization in the oropharynx^23^. At least in elderly patients with many complications, malnutrition that causes immune dysfunction may increase the risk for Candida colonization. Future studies clarifying the impact of BMI or malnutrition on the development of EC should be conducted.

Esophageal hiatal hernia and GERD, both of which are often observed in obese patients, create an acidic environment in the esophagus. Therefore, we presumed that these pathologies increase antifungal activity in the esophageal mucosa and reduced the risk of EC; however, neither factor was reduced the risk of EC development in the present study. Therefore, it is likely that the absolute amount of gastric acid secretion in the stomach may be more critical for developing EC than the backflow of acid into the esophagus. To examine the impact of acid levels on the development of EC, the pH levels in both the stomach and esophagus should be evaluated in future studies.

Our study has several limitations. First, this was a retrospective study and we could not exclude bias that affects the development of EC. Second, the immune status of the enrolled patients was not fully assessed. The patients were assessed at a community hospital and none presented with readily identifiable immunodeficiency; however, immune activity and the presence of HIV infection were not examined. Finally, we did not evaluate the degree of atrophic mucosa in patients with atrophic gastritis and the status of *H. pylori* infection (naïve, present infection, or post eradication) has not been considered. Further investigation regarding these concerns could help us to identify the risk factors for EC in more detail.

In conclusion, atrophic gastritis was found to be an independent risk factor for the development of EC among immunocompetent patients in a community hospital. The risk in these patients was further increased when combined with PPI use. We hypothesize that this is due to the synergistic decrease in acid production that occurs in atrophic gastritis and is induced by PPIs. Immune inhibition by PPIs may also play a role in allowing Candida to flourish in this environment. PPIs, which strongly inhibit gastric acid secretion, are used extensively worldwide to treat acid-related disorders such as peptic ulcer and GERD. However, 25–75% of PPI use has been reported to be inappropriate^24^. Especially, it might be required caution when we use PPI for patients with atrophic gastritis, advanced gastric cancer or post-gastrectomy.

## Conclusion

PPI use, atrophic gastritis, advanced gastric cancer and post-gastrectomy are critical risk factors for the development of EC. We suggest close monitoring for EC development when PPIs are administered to patients with these factors.

## Abbreviations

AIDS: Acquired immune deficiency syndrome
BMI: Body mass index
*C. albicans*: *Candida albicans*
CI: Confidence interval
EC: Esophageal candidiasis
GERD: Gastroesophageal reflux disease
HIV: Human immunodeficiency virus
*H. pylori*: *Helicobacter pylori*
OR: Odds ratio
PPI: Proton pump inhibitors
